# Increased amputation risk with canagliflozin treatment: behind the large cardiovascular benefit?

**DOI:** 10.1186/s12933-017-0611-x

**Published:** 2017-10-12

**Authors:** Atsushi Tanaka, Koichi Node

**Affiliations:** Department of Cardiovascular Medicine, Saga University, 5-1-1 Nabeshima, Saga, 849-8501 Japan

## Abstract

A growing body of evidence suggests that sodium-glucose cotransporter 2 (SGLT2) inhibitors appear to be a powerful option to improve the cardiovascular (CV) prognosis in high CV-risk patients with type 2 diabetes. Despite a significant reduction in major adverse CV events with SGLT2 inhibitor treatment, however, an unexpected increased risk of amputation was observed in the CANVAS program and the subsequent pharmacovigilance analysis. Although the underlying mechanisms are currently unknown, because amputation has a large negative impact on patient clinical course, clinicians want to know the exact reason for the increased amputation in the canagliflozin treatment. We herein discuss a need to elucidate the actual reasons with more appropriate statistical consideration, taking into account individual clinical course potentially involved in the diabetes-related amputation. Decreases in the hardendpoints by canagliflozin might result in an alternate increase in the other diabetes-related complications, including amputation. In addition, if amputation occurred after stopping canagliflozin, the incidence might be caused by worsened glycemic control and a decrease in hematocrit, accompanied by a subsequent worsening of diabetic foot disease. More detailed approach considering individual clinical course potentially involved in the amputation, would help to further unravel the cause for suspected risk of amputation with canagliflozin.

A great deal of clinical interest was generated by the EMPA-REG OUTCOME trial, which evaluated the cardiovascular (CV) safety of empagliflozin, a sodium-glucose cotransporter 2 (SGLT2) inhibitor, in patients with type 2 diabetes (T2D) and high CV risk [1]. Based on the potential multiple beneficial effects of SGLT2 inhibitors on the CV and renal systems [2], the results of subsequent studies with SGLT2 inhibitors have received much attention. As expected, the CANVAS program [3] revealed recently that canagliflozin also improved CV and renal outcomes in a population similar to the EMPA-REG OUTCOME trial. These studies highlighted a promising clinical application of SGLT2 inhibitors for high-quality CV and diabetes care, and suggested that these agents should play an important role in the improvement of clinical outcomes in patients with T2D.

Accumulating evidence has demonstrated that canagliflozin treatment can achieve favorable long-term improvement in glycemic and metabolic parameters and is well tolerated as either mono-therapy or in combination with other anti-hyperglycemic agents, including insulin [4–8]. Furthermore, Januzzi et al. [9] recently reported that canagliflozin attenuated the serial escalation of serum levels of N-terminal pro-brain natriuretic peptide and high-sensitivity troponin I, relative to placebo, over a 104-week period in older patients with T2D. These multi-factorial benefits obtained from canagliflozin treatment may support, at least in part, the improvement of CV outcomes observed in the CANVAS program.

Conversely, due to their unique mode of action, SGLT2 inhibitors have several signature adverse effects, such as genital/urinary tract infection, and such adverse effects were observed in both CV safety trials. In contrast, in the CANVAS program an unexpected increase (approximately twofold) in the risk of lower-extremity amputation (minor: 71%, major: 29%) was observed in the canagliflozin-treated group [3]. Most recently, Fandini et al. [10] reported that canagliflozin was associated with a higher risk of amputation relative to empagliflozin and dapagliflozin in a pharmacovigilance analysis using the US Food and Drug Administration Adverse Event Reporting System. As the authors noted, the records may not be sufficient to explain a precise causal relationship between canagliflozin exposure and amputation [10]. Furthermore, the underlying mechanisms are currently unknown, and whether the amputation risk is specific to canagliflozin remains to be elucidated. However, because amputation has a large negative impact on patient clinical course, we clinicians want to know the exact reason for the increased incidence of amputation with canagliflozin treatment.

Although the risk of lower-extremity amputation in patients with T2D has declined substantially during the past couple of decades [11, 12], globally, diabetic foot disease (DFD) is still a large clinical and socioeconomic concern [13, 14]. In patients with DFD, diabetes itself and CV pathophysiology are also likely to be of advanced clinical status. Hence, amputation risk is inherently increased in diabetes, and a history of DFD and amputation are risk factors for CV events, including re-amputation, and poorer prognosis [15, 16]. Results of a population-based cohort study showed that the risk of amputation was particularly high during the first 6 months following revascularization for critical limb ischemia [17]. Actually, a history of amputation and DFD were also strong and independent risk factors for amputation in the CANVAS program [3]. On the other hand, in a subgroup analysis of patients stratified by disease history, risk reduction in pre-defined major adverse CV events (MACE) in patients with a history of amputation was greater than in those without. Furthermore, according to the early separation of development of MACE between SGLT2 inhibitor treatment and placebo in the two trials, clinical benefits appear relatively early in the course of SGLT2 inhibitor intervention. Meanwhile, amputation in the CANVAS program occurred more often during the late phase of the study period. Therefore, it may be speculated that a favorable clinical course of reduction in MACE might influence the increased incidence of amputation, indicating a possibility that an alternate risk of amputation potentially increased in the canagliflozin-treated population who received the benefits in MACE. It may be plausible that a risk-shift to diabetes-related soft-complications, including amputation, has been raised by canagliflozin treatment. Thus, we should recognize that in the CV safety trials, the incidence of each event in the placebo group represents the natural history of the participants, while that in the specific treatment group shows the test agent-modified clinical course.

Although it may not be appropriate to compare the results of separate studies, we are left to question why the risk of amputation was increased in the CANVAS program, but not in the EMPA-REG OUTCOME trial [18]. Focusing on the differences in the other clinical characteristics between the two trials may provide a clue as to factors potentially contributing to the increase in the frequency of amputation. The EMPA-REG OUTCOME trial population was receiving secondary prevention for CV disease, while the CANVAS program population contained patients receiving both primary and secondary prevention. However, the prevalence of peripheral artery disease at baseline was almost similar (approximately 20%) between the two studies, possibly having less impact. Most recently, a real-world retrospective cohort study in which patients with T2D and established CV disease were also included (approximately 20%) demonstrated that no increased signal of incidence of below-knee lower extremity amputation was observed in new users of canagliflozin compared to other SGLT2 inhibitors and anti-hyperglycemic agents [19]. This may indicate a possibility that the risk of amputation documented in the CANVAS program resulted, in part, from variance in other factors between the trials.

We next noticed that adverse events related to volume depletion occurred more often in the canagliflozin-treated group than in the placebo group, despite a similar incidence in the EMPA-REG OUTCOME trial, which might accordingly contribute to the circulatory failure in the distal peripheral arterial beds. However, diuresis and subsequent volume depletion would be more evident at the early phase of SGLT2 inhibitor treatment, inconsistent with the phase of increased incidence of amputation.

With regard to adherence to the study drugs, a total of 25% of the participants in the EMPA-REG OUTCOME trial and 29% in the CANVAS program discontinued their assigned treatment. In the EMPA-REG OUTCOME trial, the incidence of stroke showed a higher tendency in the analysis of the intent-to-treat population than in the on-treatment analysis. When administration of an SGLT2 inhibitor is stopped, several SGLT2 inhibitor-mediated favorable effects on metabolic and hemodynamic parameters might be eliminated (e.g., worsened glycemic control and decrease in hematocrit levels), followed by progression of distal peripheral arteriosclerosis prone to ischemia and foot ulcer, leading to possible increases in amputation. Although it is still not easy to determine or explain the specific reason for an increased frequency of amputation with canagliflozin treatment, more detailed statistical approaches considering the individual clinical course potentially involved in diabetes-related amputation should further help us to understand why and how its risk increased in the canagliflozin-treated population.

For clinicians, especially in the cardiovascular field, the emerging SGLT2 inhibitors should be a promising option to improve CV outcomes in the care of patients with T2D at high CV risk. If possible reason(s) for the increased risk of amputation with canagliflozin treatment are uncovered behind the large CV benefit, our understanding of SGLT2 inhibitors would be much better for providing effective and safe CV-diabetes care. The ultimate goal is to exploit the CV benefit of SGLT2 inhibitors, while avoiding adverse effects, for appropriate patients who need SGLT2 inhibitor-mediated CV benefits, because SGLT2 inhibitor treatment in the recent landmark studies markedly reduced the incidence of MACE paramount to the clinical outcomes.

## Abbreviations

CV: cardiovascular
DFD: diabetic foot disease
MACE: major adverse CV events
SGLT2: sodium–glucose cotransporter 2
T2D: type 2 diabetes
